# In Vitro Evaluation of the Effects of Cadmium on Endocytic Uptakes of Proteins into Cultured Proximal Tubule Epithelial Cells

**DOI:** 10.3390/toxics8020024

**Published:** 2020-04-01

**Authors:** Hitomi Fujishiro, Hazuki Yamamoto, Nobuki Otera, Nanae Oka, Mei Jinno, Seiichiro Himeno

**Affiliations:** Laboratory of Molecular Nutrition and Toxicology, Faculty of Pharmaceutical Sciences, Tokushima Bunri University, Tokushima 770-8514, Japan; donai-do@ph.bunri-u.ac.jp (H.F.);

**Keywords:** kidney, endocytosis, β_2_-microglobulin, metallothionein, flow cytometry, proximal tubule epithelial cells

## Abstract

Cadmium (Cd) is an environmental pollutant known to cause dysfunctions of the tubular reabsorption of biomolecules in the kidney. Elevated levels of urinary excretion of low-molecular-weight proteins such as β_2_-microglobulin (β_2_-MG) have been used as an indicator of Cd-induced renal tubular dysfunctions. However, very few studies have examined the direct effects of Cd on the reabsorption efficiency of proteins using cultured renal cells. Here, we developed an in vitro assay system for quantifying the endocytic uptakes of fluorescent-labeled proteins by flow cytometry in S1 and S2 cells derived from mouse kidney proximal tubules. Endocytic uptakes of fluorescent-labeled albumin, transferrin, β_2_-MG, and metallothionein into S1 cells were confirmed by fluorescence imaging and flow cytometry. The exposure of S1 and S2 cells to Cd at 1 and 3 µM for 3 days resulted in significant decreases in the uptakes of β_2_-MG and metallothionein but not in those of albumin or transferrin. These results suggest that Cd affects the tubular reabsorption of low-molecular-weight proteins even at nonlethal concentrations. The in vitro assay system developed in this study to evaluate the endocytic uptakes of proteins may serve as a useful tool for detecting toxicants that cause renal tubular dysfunctions.

## 1. Introduction

Cadmium (Cd) is an environmental pollutant that causes renal toxicity in animals and humans after chronic exposure in the diet [1]. Due to both the high affinity of Cd for sulfhydryl moieties in biomolecules within cells and the difficulty of excretion from cells, the biological half-life of Cd in the human kidney has been calculated to be more than 25 years [2]. The renal accumulation of Cd results in characteristic renal toxicity is known as Fanconi syndrome at the advanced stage [3,4,5]. Cd accumulation in the proximal tubules of the kidney has been believed to disturb the reabsorption of the luminal biomolecules, which are filtered through the glomerulus into proximal tubule epithelial cells (PTECs). Animals and humans exposed to Cd for a long time show increased urinary excretion of glucose, amino acids, and low-molecular-weight (LMW) proteins such as β_2_-microglobulin (β_2_-MG) and metallothionein (MT) [6,7,8]. Enhanced urinary levels of β_2_-MG have been used as sensitive and reliable indicators of Cd-induced renal tubular damage [9,10].

The reabsorption of luminal biomolecules including β_2_-MG and MT by PTECs is mediated by megalin-dependent endocytosis at the apical membrane of PTECs [11,12,13,14]. However, many studies on Cd cytotoxicity have focused on the mechanisms of cell lethality, including apoptosis caused by Cd [15,16,17], and only a few studies have examined Cd’s direct effects on the efficiency of protein reabsorption by PTECs [18,19], especially under conditions where PTECs are surviving in the presence of Cd.

Recently, we developed an in vitro experimental system using mouse PTEC-derived S1, S2, and S3 cells, which maintain fundamental features of S1, S2, and S3 segment-specific expression of genes including metal transporters [20,21]. In the present study, we attempted to develop an in vitro experimental system for evaluating the endocytosis efficiency of LMW and high-molecular-weight (HMW) proteins into S1 and S2 cells derived from the S1 and S2 segments of proximal tubules where the reabsorption of glomerular-filtered proteins is highly active. To visualize and quantify the amounts of endocytosed proteins, we used fluorescent-labeled albumin, transferrin, β_2_-MG, and MT. Here, we show that flow cytometric analyses of the incorporation of fluorescent-labeled proteins into cultured PTECs can be used to quantitatively evaluate endocytosis efficiency. By using this in vitro assay system, we detected decreases in the endocytic uptakes of β_2_-MG and MT in cultured PTECs exposed to Cd.

## 2. Materials and Methods

### 2.1. Materials

Mouse anti-megalin monoclonal antibody was purchased from Abcam (Cambridge, MA, USA). Goat anti-cubilin polyclonal antibody was purchased from Santa Cruz Biotechnology (Dallas, TX, USA). Rabbit anti-transferrin receptor polyclonal antibody was purchased from Abnova (Taipei, Taiwan). Rabbit anti-β-actin polyclonal antibody, rabbit anti-Early Endosome Antigen 1 (EEA1) antibody, anti-rabbit IgG HRP-linked antibody, anti-mouse IgG HRP-linked antibody, and anti-goat IgG HRP-linked antibody were purchased from Cell Signaling Technology (Danvers, MA, USA). Alexa 555 anti-rabbit IgG antibody was purchased from Invitrogen (Carlsbad, CA, USA). Albumin-fluorescein isothiocyanate conjugate was purchased from Sigma-Aldrich (St. Louis, MO, USA), and Alexa Fluor^®^ 488-conjugated ChromPure Mouse Transferrin was purchased from Jackson ImmunoResearch Laboratories (West Grove, PA, USA). Immortalized human renal proximal tubular epithelial cells (hRPTECs: CRL-4031) was obtained from ATCC (American Type Culture Collection, Manassas, VA, USA).

### 2.2. Cell Culture

S1, S2 cells, and hRPTEC were cultured in Dulbecco’s modified Eagle’s medium/Ham’s Nutrient Mixture F12 supplemented with 5% fetal bovine serum (FBS), 1 μg/mL insulin, 10 ng/mL epidermal growth factor, 10 μg/mL transferrin, and penicillin/streptomycin under 5% CO_2_ at 37 °C, as described previously [20]. Cells were used at the passages of 3–10 from the stocked original cells.

### 2.3. Purification of Recombinant Proteins and Their Fluorescent Labeling

The cloned mMT-I/pGEX-4T-1 plasmid and mouse β_2_-MG/pGEX-4T-1 plasmid were transformed into BL21(DE3)pLysS (Promega, Madison, WI, USA). The selected transformed cells were grown in 10 mL SOB medium containing 50 μg/mL ampicillin for 16 h at 37 °C until the optical density at 600 nm reached 0.3–0.4. The expression of MT and β_2_-MG proteins was induced by incubation with 1 mM IPTG for 6 h at 37 °C. The cultured cells were harvested by centrifugation at 8000 rpm for 10 min at 4 °C, and the GST-fusion proteins were purified by using MagneGSTTM Protein Purification (Promega). After a dialysis against PBS, the GST-fusion MT and GST-fusion β_2_-MG proteins were digested with thrombin (GE Healthcare, Buckinghamshire, UK). The lysates were loaded onto GST GraviTrapTM gravity-flow columns (GE Healthcare) to remove GST proteins, and the lysates were loaded onto a Benzamidine Sepharose 4 Fast Flow resin (GE Healthcare) to remove thrombin.

The purified recombinant MT and β_2_-MG proteins were conjugated with fluorescein isothiocyanate (FITC) by Fluorescein Labeling Kit-NH2 (Dojindo, Kumamoto, Japan). For FITC-labeled albumin and Alexa-labeled transferrin, commercially available albumin-fluorescein isothiocyanate conjugate and Alexa Fluor^®^ 488-conjugated ChromPure Mouse Transferrin, respectively, were used.

### 2.4. Fluorescence Imaging of the Labeled Proteins in S1 Cells

S1 cells grown in glass-bottom dishes were incubated with each fluorescent-labeled protein and Hoechst33258 for 30 min, washed with phosphate-buffered saline (PBS), fixed with 4% paraformaldehyde in PBS for 5 min on ice, and then permeabilized with 0.5% TritonX-100 in PBS for 15 min at room temperature.

For the immunostaining of EEA1 to detect the early endosome, the fixed cells were washed with PBS and incubated with a blocking buffer containing bovine serum albumin (BSA) in PBS for 0.5 h at room temperature. The cells were then incubated with an anti-EEA1 antibody at a 1:100 dilution in blocking buffer for 1 h at room temperature. After washing with PBS, the cells were incubated with Alexa 555 anti-rabbit IgG at a 1:500 dilution in blocking buffer for 1 h at room temperature.

The distributions of FITC-albumin and Alexa-transferrin were visualized by a Nikon A1R-Si HD confocal microscope (Nikon, Tokyo, Japan), and those of FITC-MT and β_2_-MG were visualized by a BZ-X700 all-in-one fluorescence microscope (Keyence, Osaka, Japan).

### 2.5. Assay for Sensitivity to Cd

Cells were plated on 96-well plates at a density of 3 × 10^3^–2 × 10^4^ cells per well, incubated for 24 h in Cd-free medium, and then treated with CdCl_2_ for 1, 3, or 6 days. The media were not changed during the Cd exposure period. The alamarBlue^®^ assay (Invitrogen) was used to determine cell viability. AlamarBlue solution was premixed with fresh medium and added to the 96-well plates. After incubation for 2 h, the reduction in alamarBlue by active cells was determined by absorbance at 540 nm and expressed as the percentages compared to that of control cells.

### 2.6. Measurement of Endocytosis Efficiency by Flow Cytometry

Endocytosis efficiency was determined by using flow cytometry (Guava easyCyte 6HT/2L; Millipore, Billerica, MA, USA). S1 and S2 cells (1 × 10^5^ cells in 6-well dishes) were cultured with Cd at the concentrations of 10 or 15 µM for 1 day, 1 or 3 µM for 3 days, and 0.1 or 0.5 µM for 6 days. hRPTECs were cultured with Cd at the concentrations of 5 or 25 M for 3 days. After the media were changed to Cd- and serum-free ones, the cells were incubated with each fluorescent-labeled protein for 30 min, washed three times with 0.5 mL ice-cold PBS, and then harvested and subjected to flow cytometry. In order to quantify the percentages of cell populations incorporating fluorescent proteins, the populations were divided into quadrants, and the percentages of the cell populations in the lower-right section were calculated.

### 2.7. Immunoblot Analysis

Cells were harvested with a lysis buffer, and the extracted proteins were separated by SDS polyacrylamide gel electrophoresis (7.5–10%) and electrophoretically transferred to a polyvinylidene fluoride membrane. The transblots were preincubated with 5% nonfat dry skim milk or BSA in Tris-buffered saline (TBS, pH 7.4) and then incubated overnight with the antibody against each protein. After washing with TBS/0.05% Tween 20, the membranes were incubated with either anti-rabbit, anti-mouse, or anti-goat IgG HRP-linked antibody (1:3000). The membrane was rinsed with TBS/0.05% Tween 20, and the immunoreactive bands were developed by ECL systems (Millipore Billerica, MA, USA).

### 2.8. Statistical Analysis

Statistically significant differences were determined by one-way ANOVA followed by Bonferroni multiple comparisons using a Statcel 3 software (ver. 3, OMS Publication, Saitama, Japan, 2012).

## 3. Results

### 3.1. Fluorescence Imaging of Endocytic Uptakes of the Labeled Proteins into Mouse PTECs

**Figure 1:**
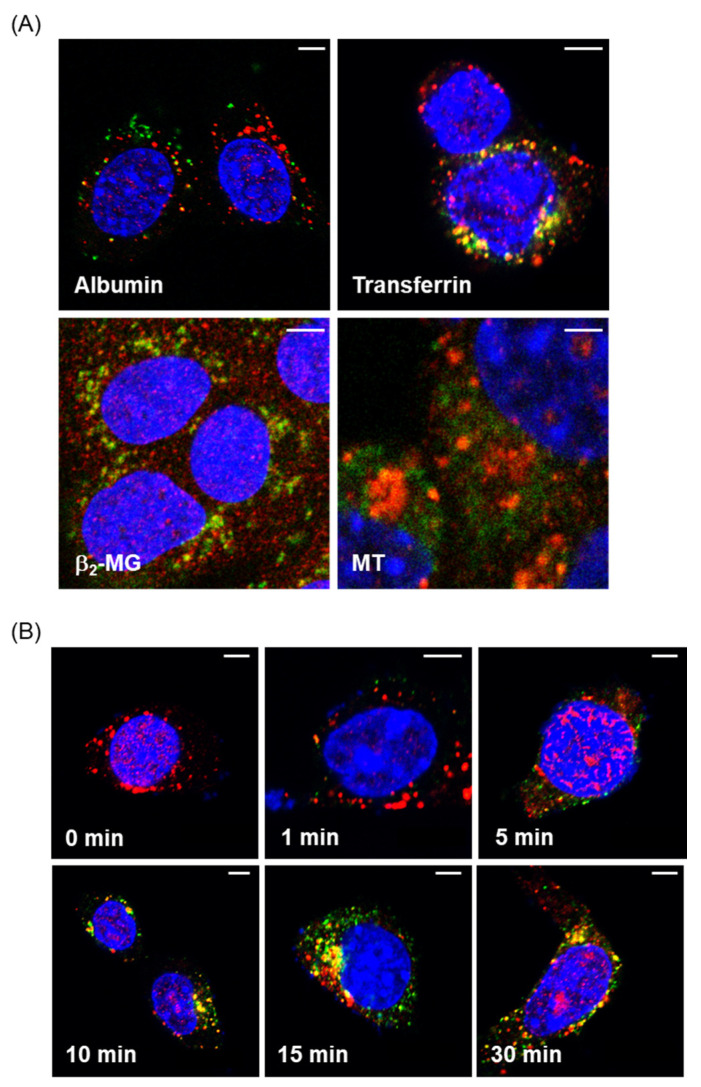
Fluorescence imaging of endocytosed proteins in mouse proximal tubule epithelial cell (PTEC)-derived S1 cells. (**A**) S1 cells were incubated with 50 µg/mL fluorescein isothiocyanate (FITC)-albumin, Alexa-transferrin, FITC-β_2_-MG, or FITC-MT (green) for 30 min and then fixed with paraformaldehyde for immunofluorescence labeling with anti-EEA1 (red). Yellow staining demonstrates the colocalization of fluorescent-labeled proteins and early endosomes. (**B**) S1 cells were incubated with Alexa-transferrin for 1, 5, 10, 15, and 30 min. The localization of Alexa-transferrin (green) and early endosomes stained with anti-EEA1 (red) was visualized by confocal microscopy. Bars, 5 µm.

We first confirmed the incorporation of fluorescent-labeled proteins into mouse PTECs with confocal and fluorescence microscopes. As shown in Figure 1A, the green fluorescent signals of FITC and Alexa488 were clearly detected in S1 cells 30 min after the addition of FITC-labeled albumin, β_2_-MG, MT, and Alexa-labeled transferrin. The yellow fluorescent signals observed in the cells indicate the overlapping of the red fluorescence of the antibody against EEA1 and the green fluorescence of the proteins, suggesting the incorporation of the labeled proteins into the early endosomes.

Figure 1B shows the time-dependent changes in the fluorescent signals of transferrin that showed the strongest fluorescent intensities. The green signals of transferrin began to be detected within cells 1 min after the addition of the protein. Yellow fluorescent signals, indicative of the incorporation of transferrin into the early endosomes, began to appear at 10 min and then increased up to 30 min. Thus, the results of the fluorescence imaging provided evidence for endocytic incorporation of the labeled proteins into S1 cells.

### 3.2. Quantification of Endocytic Uptakes of the Labeled Proteins into Mouse PTECs

**Figure 2:**
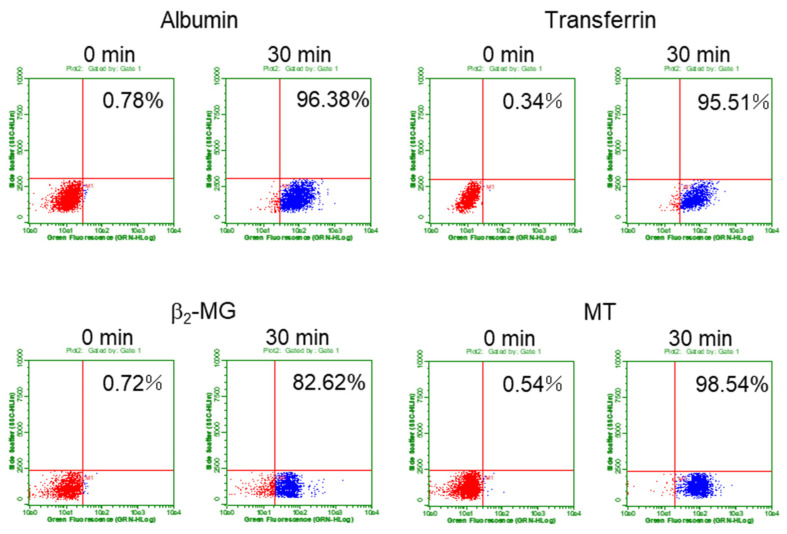
Evaluation of endocytosis efficiency of the fluorescent-labeled proteins by using flow cytometry. The cells were cultured with each of the fluorescent-labeled protein for 0 or 30 min and applied to flow cytometry. Typical quadrant data of each protein in S1 cells were shown here. X-axis indicates fluorescent intensity and y-axis indicates side scattering. The untreated cells (0 min) showing the auto-fluorescence were gated to be the lower-left section in the quadrants. The cell populations in the lower-right section were expressed as the percentage of total cells and used as the indicator of endocytosis efficiency (%) in the subsequent experiments.

Next, we set up a quantification system for determining the endocytosis efficiency of each protein into the cells by using flow cytometry. The cells were cultured with each fluorescent-labeled protein for 30 min, washed, harvested, and applied to flow cytometry. As shown in Figure 2, the percentages of the cell population in lower-right section in the quadrants were used as the indicator of endocytosis efficiency (%). Based on the results of preliminary experiments, the amounts of the labeled proteins were decided to be 25 µg/mL for albumin and transferrin and 50 µg/mL for β_2_-MG and MT as the optimal conditions for incorporation. We used both S1 and S2 cells derived from the S1 and S2 segments of mouse proximal tubules, respectively, since both cell lines showed similar expression levels of megalin, cubilin, and transferrin receptor, which are essential for endocytosis in PTECs (Figure S1). We determined the time-dependent changes in endocytosis efficiency for each protein (Figures S2 and S3). Although these data are obtained by preliminary experiments, it was shown that the uptake rates of albumin and transferrin during 30 min were almost the same between S1 and S2 cells while those of β_2_-MG and MT were lower into S2 cells than into S1 cells. Since most proteins showed maximal uptakes at 30 min, the effects of Cd exposure on the endocytic uptakes of these proteins were examined 30 min after the addition of the labeled proteins in the subsequent experiments.

The high efficiency of transferrin incorporation into S1 and S2 cells may be partially caused by the expression of transferrin receptor in these cells (Figure S1). It is known that the transferrin receptor in PTECs is expressed in the basolateral [22] and apical [23] membranes, whereas megalin and cubilin are expressed at the apical membrane [24], suggesting that both uptake systems for transferrin contribute to the highly efficient uptake into the endosomes in S1 cells.

### 3.3. Effects of Cd Exposure on the Endocytic Uptakes of the Labeled Proteins into Mouse PTECs

**Figure 3:**
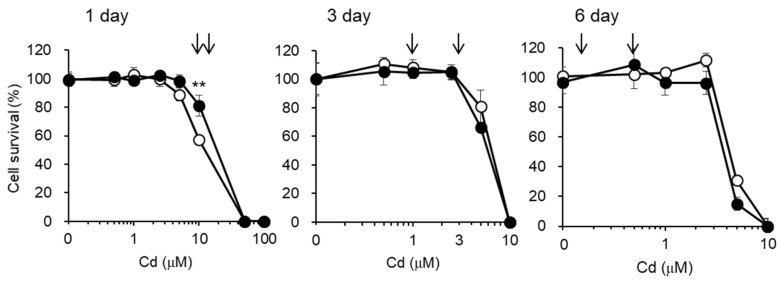
Cytotoxicity of Cd in S1 and S2 cells. S1 (open circles) and S2 (closed circles) cells cultured in 96-well plates were incubated with the indicated concentrations of CdCl_2_ for 1, 3, and 6 days. Cell viability was determined by alamarBlue^®^ assay and expressed as a percentage of the nontreated cells. From these results, the Cd concentrations to be used in the subsequent experiments were determined (arrows). Data are presented as means ± SD (*n* = 4–6). Statistically significant difference between S1 and S2 cells was indicated as ** *p* < 0.01.

Before examining the effects of Cd exposure on the endocytic uptakes of the labeled proteins into S1 and S2 cells, we checked the lethal toxicity of Cd in S1 and S2 cells using the alamarBlue assay (Figure 3). Based on the results of this assay, we selected sublethal doses of Cd, as indicated by the arrows in Figure 3, for the subsequent endocytosis experiments. We also attempted to use much higher doses of Cd (5 µM Cd for 3 days and 1 µM Cd for 6 days), but S1 and S2 cells could not survive these concentrations of Cd when cultured in 6-well plates for endocytosis experiments. The discrepancy in cytotoxicity between the 96-well (alamarBlue^®^ assay) and 6-well plates may be attributable to the differences in cell density. Therefore, in the endocytosis experiments we used 10 and 15 µM Cd for 1 day, 1 and 3 µM Cd for 3 days, and 0.1 and 0.5 µM Cd for 6 days. Under these conditions, very few cells were found to be detached from the plates at the end of Cd exposure, and the three-times washing of the cells with ice-cold PBS before harvesting did not result in the detachment of the cells. Thus, the effects of Cd on the endocytosis efficiencies in the following experiments were carried out with the cells including least populations of dead cells.

**Figure 4:**
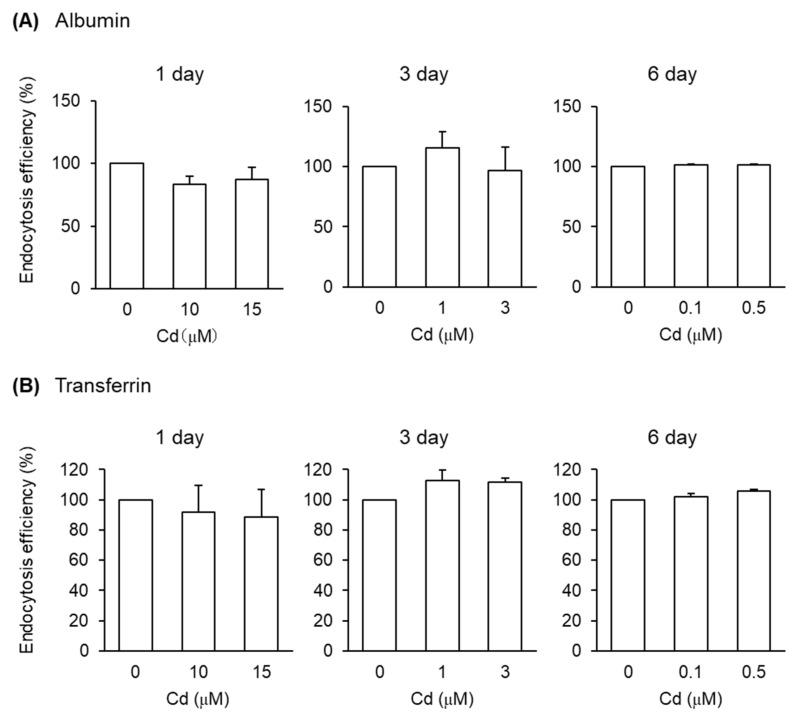
Effects of Cd on the endocytosis efficiencies of albumin and transferrin into S1 cells. S1 cells were exposed to CdCl_2_ for 1, 3, and 6 days and then incubated with FITC-albumin (**A**) or Alexa-transferrin (**B**) for 30 min. The endocytosis efficiencies were determined by flow cytometry and expressed as percentages of the control cells (no exposure to Cd). Data are presented as means ± SD (*n* = 3–4).

**Figure 5:**
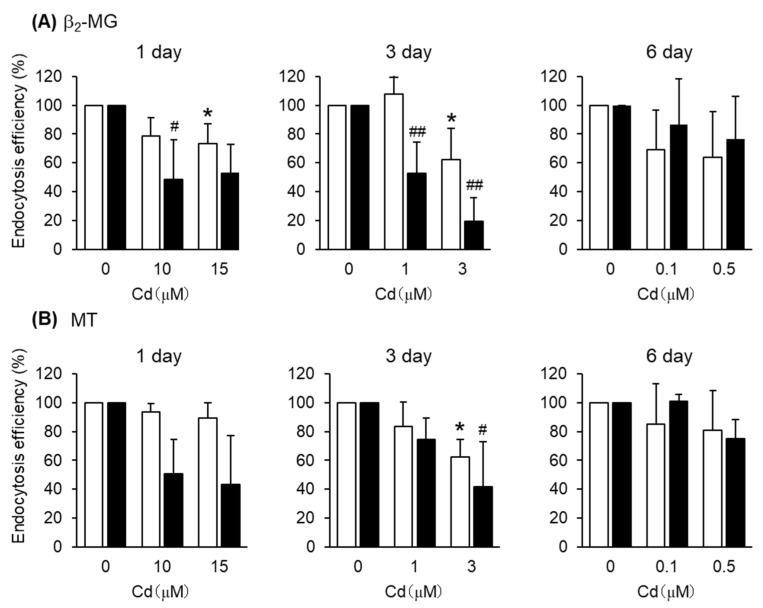
Effects of Cd on the endocytosis efficiencies of β_2_-MG and MT into S1 and S2 cells. S1 and S2 cells were exposed to CdCl_2_ for 1, 3, and 6 days and then incubated with FITC-β_2_-MG (**A**) or FITC-MT (**B**) for 30 min. The endocytosis efficiencies were determined by flow cytometry and expressed as percentages of the control cells (no exposure to Cd). Open and closed columns represent S1 and S2 cells, respectively. Data are presented as means ± SD (*n* = 3–4). Statistical significance of the dose dependence determined by one-way ANOVA was detected in the following settings: day1-S1 cells (*p* < 0.05), day1-S2 cells (*p* < 0.05), day3-S1 cells (*p* < 0.05), and day3-S2 cells (*p* < 0.01) for β_2_-MG (**A**), and day3-S1 cells (*p* < 0.05) and day3-S2 cells (*p* < 0.05) for MT (**B**). Statistical significances compared with the control cells determined by Bonferroni multiple comparisons are indicated as * *p* < 0.05, ** *p* < 0.01 (S1 cells) and # *p* < 0.05, ## *p* < 0.01 (S2 cells).

After the exposure of S1 cells to Cd at these concentrations for 1, 3, and 6 days, the endocytic uptakes of the labeled albumin and transferrin into S1 cells were examined (Figure 4 and Figure S4). However, Cd exposure did not affect the endocytic uptake of either albumin or transferrin. On the other hand, the endocytic uptakes of β_2_-MG and MT were affected by Cd exposure depending on the exposure duration (Figure 5). The 1- and 3-day exposures of S1 and S2 cells to Cd resulted in statistically significant decreases in the endocytic uptake of β_2_-MG (Figure 5A), whereas only the 3-day exposure to Cd resulted in statistically significant decreases in endocytic uptakes of MT (Figure 5B and Figure S4). The 6-day exposure to Cd did not cause any significant decreases in endocytic uptakes of either β_2_-MG or MT in either cells.

### 3.4. Effects of Cd Exposure on the Endocytic Uptakes of the Labeled Proteins into Human PTECs

**Figure 6:**
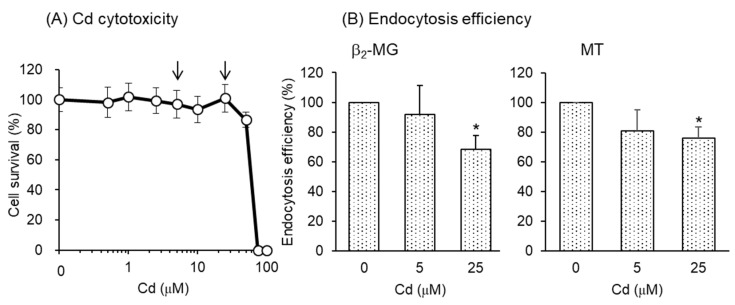
The effects of Cd exposure for 3 days on the endocytosis efficiencies of β_2_-MG and MT into hRPTEC human renal cells. (**A**) Cell viability was determined by alamarBlue assay and expressed as the percentages of the nontreated cells. From these results, the Cd concentrations to be used in the endocytosis experiment were determined (arrows). Data are presented as means ± SD (*n* = 4–6). (**B**) The cells were exposed to CdCl_2_ for 3 days and then incubated with FITC-β_2_-MG or FITC-MT for 30 min. The endocytosis efficiencies were determined by flow cytometry and expressed as percentages of the control cells (no exposure to Cd). Data are presented as means ± SD (*n* = 3–4). Statistical significance of the dose dependence determined by one-way ANOVA was detected in both β_2_-MG (*p* < 0.05) and MT (*p* < 0.05). Statistical significance compared with the control cells determined by Bonferroni multiple comparisons are indicated as * *p* < 0.05.

To test whether Cd exposure also affects endocytic uptakes of β_2_-MG and MT in human PTECs, we utilized hRPTECs, an immortalized cell line derived from human kidney PTECs. Since the effects of Cd on the endocytic uptakes of β_2_-MG and MT in mouse S1 and S2 cells were clearly detected after the 3-day exposure to Cd (Figure 5), hRPTECs were exposed to Cd for 3 days. Prior to the endocytosis experiment, we checked the sensitivity of hRPTECs to Cd. As shown in Figure 6A, hRPTECs were highly resistant to Cd compared with S1 or S2 cells. Therefore, we used 5 and 25 µM Cd for endocytosis experiments in hRPTECs. As shown in Figure 6B, the endocytic uptakes of both β_2_-MG and MT were significantly reduced by 3-day exposure to Cd.

## 4. Discussion

Disturbances in the tubular reabsorption of glomerular-filtered biomolecules by PTECS in kidney are a hallmark of Cd-induced nephrotoxicity. The urinary excretion of β_2_-MG has been particularly widely used as an indicator of renal tubular dysfunction among residents of Cd-polluted areas [6,9,10]. However, the precise mechanisms underlying Cd-induced dysfunctions of tubular reabsorption of LMW proteins by PTECs have not been fully investigated. This is partly due to the lack of a proper in vitro experimental system.

In the present study, we developed an in vitro experimental system for evaluating the endocytosis efficiency of fluorescent-labeled proteins into cultured PTECs derived from mouse and human kidney. The endocytic uptakes of the labeled proteins and their cellular localization were confirmed by fluorescence imaging (Figure 1). Flowcytometric analyses of the fluorescent-labeled proteins have enabled us to quantitatively evaluate the uptake rates of labeled proteins into S1 and S2 cells (Figure 2). The exposure of these cells to sublethal doses of Cd for 3 days resulted in significant decreases in the endocytic uptakes of β_2_-MG and MT (Figure 5), but not in those of albumin or transferrin (Figure 4). These results demonstrated that the assay system developed in this study permitted the detection of Cd-induced declines in renal reabsorption of LMW proteins in cultured PTECs. The reason for the absence of the effects of Cd after 6-day exposure remains unknown. Possibly, more complicated factors are involved in the 6-day exposure than the 3-day exposure experiments. Although future studies are required for the mechanisms of Cd-induced decreases in the LMW protein uptakes, this assay system may be useful for screening other renal toxicants that may cause damage in tubular reabsorption.

To date, epidemiological studies in humans and experimental studies in animals have linked increases in urinary excretion of LMW proteins such as β_2_-MG with the loss of functional PTECs and nephrons in the kidney at the advanced stage of Cd nephrotoxicity [25,26]. Most mechanistic studies on Cd cytotoxicity have focused on the molecular pathways leading to Cd-associated cell death and not on the direct effects of Cd on the endocytic uptakes of LMW proteins in living PTECs [15,16,17]. However, the effects of moderately higher, but not lethal, doses of Cd on the reabsorption efficiencies of LMW proteins by the surviving PTECs remain unclear. The results of this study demonstrated that a 3-day exposure to Cd resulted in significant declines in the endocytic uptakes of β_2_-MG and MT under the conditions in which nonlethal doses of Cd were used. This could not be ascribed simply to the increase in dead cells after Cd exposure, since no effects were observed in the endocytosis efficiencies of albumin or transferrin (Figure 4) and only the surviving cells that were not detached from the plates during Cd exposure were used for flow cytometry analyses. Although a few studies have investigated the effects of Cd on the interactions of LMW proteins with megalin/cubilin systems in cultured renal cells [18,19], the present study utilized flow cytometry for quantitatively evaluating the endocytosis efficiency of the proteins and showed the effects of Cd on the uptakes of LMW proteins in cultured PTECs.

Although many epidemiological studies undertaken in Cd-polluted areas have demonstrated that urinary excretion of β_2_-MG is an excellent biomarker for renal tubular dysfunctions [6,9,10], recent evidence suggested that β_2_-MG plays much broader roles as a biomarker not only for tubular dysfunctions, but also for glomerular dysfunctions as well as non-renal diseases [27]. The results of our in vitro study added a piece of evidence that the decreased incorporation of β_2_-MG into renal tubular cells is involved in Cd-induced kidney damages. Future studies are required to test whether the decrease in the β_2_-MG incorporation into renal tubular cells is inducible specifically by Cd, or commonly by other renal toxicants using this assay system.

Compared with the uptakes of β_2_-MG and MT, those of albumin and transferrin by S1 cells appear to be far less sensitive to Cd toxicity (Figure 4). Many human studies have suggested that the urinary excretion of β_2_-MG, a LMW protein, reflects damage in renal tubular reabsorption, whereas the urinary excretion of albumin, an HMW protein, may reflect the dysfunction of glomeruli [28,29,30], although recent evidence has indicated that tubular reabsorption of albumin should not be ignored [31]. The results of this study also demonstrated that substantial amounts of albumin can be taken up by cultured PTECs. Although the differences in the endocytic pathways between HMW albumin and LMW β_2_-MG remains unclear, the higher sensitivity of β_2_-MG than albumin in this in vitro assay system may reflect the in vivo observation that β_2_-MG is the most sensitive indicator for renal tubular damage caused by Cd exposure [28,29]. Regarding the comparisons between HMW and LMW proteins, the differences in molecular weights, i.e., albumin and MT, could have affected the endocytosis efficiency because we used 25 µg/mL albumin and 50 µg/mL MT as the optimal conditions, not based on a molar basis, in this assay.

The strengths and limitations of this study should be noted. (1) The use of flow cytometry enabled the quantitative evaluation of the endocytosis efficiencies of fluorescent-labeled proteins, (2) the use of both mouse- and human-derived PTECs enabled the confirmation of reproducibility of Cd effects, and (3) the use of sublethal doses of Cd enabled the sensitive detection of the effects of Cd on the PTECs that survived the lethal toxicity of Cd. However, the observed effects of Cd on the endocytosis efficiencies of β_2_-MG and MT were not so marked, though statistically significant, and the involvements of Cd cytotoxicity on the results of flow cytometry may not be completely excluded. Since the in vivo dysfunctions of renal tubular reabsorption generally occur under conditions where a large part of nephrons and PTECs is lost [25,26], the in vitro effects of renal toxicants on the endocytosis efficiency in the living PTECs may reflect only a part of the whole events of renal dysfunction. Nevertheless, this in vitro assay system may provide a useful tool for the future screening of other renal toxicants and for more detailed mechanistic studies.

For future applications of this assay system to the detection of possible renal toxicants damaging tubular reabsorption, the merits and disadvantages of this system should be discussed here. Since mouse S1 and S2 cells and human hRPTECs are all immortalized cell lines, they can provide reproducible and reliable results compared with the primary cultured PTECs prepared freshly from the proximal tubules of kidney. In this study, both mouse and human PTECs showed decreases in endocytic uptakes of β_2_-MG and MT after the 3-day exposure to Cd. However, approximately ten times higher concentrations of Cd were required in hRPTECs than in S1 and S2 cells to produce similar detrimental effects (Figure 6) due to the high Cd resistance of hRPTECs, which is the major drawback of using hRPTECs. S2 cells showed a lower efficiency of endocytic uptakes of β_2_-MG and MT than S1 cells, whereas the uptake efficiencies of albumin and transferrin were similar to those of S1 cells (Figure 2). Although both S1 and S2 cells showed significant decreases in endocytic uptakes of β_2_-MG and MT (Figure 5) when exposed to Cd for 3 days, S1 cells may be more preferable for screening renal toxicants that affect the endocytic uptakes of LMW proteins, since the uptake efficiencies of LMW proteins under unexposed conditions are stable and reliable in S1 cells. We are now planning a screening study using S1 cells to test whether other renal toxicants affect the endocytic uptakes of β_2_-MG.

## 5. Conclusions

The aim of this study was to establish an in vitro assay system for evaluating the efficiency of endocytic uptakes of LMW and HMW proteins labeled with fluorescent moiety in cultured PTECs. Flowcytometric determinations of the uptakes of the fluorescent-labeled proteins into mouse S1 and S2 cells and human hRPTECs enabled us to find that a 3-day exposure to Cd resulted decreased endocytic uptakes of β_2_-MG and MT in all these cells. Thus, these cells, especially S1 cells, proved to be useful for the elucidation of the mechanisms of Cd-induced dysfunctions of renal tubular reabsorption. In future studies, this in vitro system can be used to investigate the effects of Cd exposure on the expression of megalin and cubilin at the apical membrane, the functions of mitochondria, which provide the energy for endocytosis, and other machineries required for the process of endocytosis. This system may also serve as a tool for studying the mechanisms of other toxicants causing renal tubular dysfunctions.

## Supplementary Materials

The following are available online at https://www.mdpi.com/2305-6304/8/2/24/s1, Figure S1: Expression levels of the proteins involved in endocytosis in S1 and S2 cells, Figure S2: Time-dependent changes in endocytic uptakes of albumin, transferrin, β_2_-MG, and MT, Figure S3: Time-dependent changes in endocytic uptakes of albumin, transferrin, β_2_-MG, and MT, Figure S4: Effects of Cd on the endocytosis efficiencies of albumin and β_2_-MG into S1 cells.

## References

[1] Himeno S., Aoshima K. (2019). Cadmium Toxicity—New Aspects in Human Disease, Rice Contamination, and Cytotoxicity.

[2] Elinder C.G., Lind B., Kjellström T., Linnman L., Friberg L. (1976). Cadmium in kidney cortex, liver, and pancreas from Swedish autopsies. Estimation of biological half time in kidney cortex, considering calorie intake and smoking habits. Arch. Environ. Health.

[3] Blainey J.D., Adams R.G., Brewer D.B., Harvey T.C. (1980). Cadmium-induced osteomalacia. Br. J. Ind. Med..

[4] Takebayashi S., Jimi S., Segawa M., Kiyoshi Y. (2000). Cadmium induces osteomalacia mediated by proximal tubular atrophy and disturbances of phosphate reabsorption. A study of 11 autopsies. Pathol. Res. Pract..

[5] Savolainen H. (1995). Cadmium-associated renal disease. Ren. Fail..

[6] Chan W.Y., Rennert O.M. (1981). Cadmium nephropathy. Ann. Clin. Lab. Sci..

[7] Tohyama C., Shaikh Z.A., Ellis K.J., Cohn S.H. (1981). Metallothionein excretion in urine upon cadmium exposure: Its relationship with liver and kidney cadmium. Toxicology.

[8] Satarug S. (2018). Dietary cadmium intake and its effects on kidneys. Toxics.

[9] Ikeda M., Ezaki T., Moriguchi J., Fukui Y., Ukai H., Okamoto S., Sakurai H. (2005). The threshold cadmium level that causes a substantial increase in β_2_-microglobulin in urine of general populations. Tohoku J. Exp. Med..

[10] Shiroishi K., Kjellström T., Kubota K., Evrin P.E., Anayama M., Vesterberg O., Shimada T., Piscator M., Iwata T., Nishino H. (1977). Urine analysis for detection of cadmium-induced renal changes, with special reference to β_2_-microglobulin. A cooperative study between Japan and Sweden. Environ. Res..

[11] Klassen R.B., Crenshaw K., Kozyraki R., Verroust P.J., Tio L., Atrian S., Allen P.L., Hammond T.G. (2004). Megalin mediates renal uptake of heavy metal metallothionein complexes. Am. J. Physiol. Ren. Physiol..

[12] Christensen E.I., Birn H. (2001). Megalin and cubilin: Synergistic endocytic receptors in renal proximal tubule. Am. J. Physiol. Ren. Physiol..

[13] Sabolic I., Ljubojevic M., Herak-Kramberger C.M., Brown D. (2002). Cd-MT causes endocytosis of brush-border transporters in rat renal proximal tubules. Am. J. Physiol. Ren. Physiol..

[14] Onodera A., Tani M., Michigami T., Yamagata M., Min K.S., Tanaka K., Nakanishi T., Kimura T., Itoh N. (2012). Role of megalin and the soluble form of its ligand RAP in Cd-metallothionein endocytosis and Cd-metallothionein-induced nephrotoxicity in vivo. Toxicol. Lett..

[15] Tokumoto M., Lee J.Y., Satoh M. (2019). Transcription factors and downstream genes in cadmium toxicity. Biol. Pharm. Bull..

[16] Fujiwara Y., Lee J.Y., Tokumoto M., Satoh M. (2012). Cadmium renal toxicity via apoptotic pathways. Biol. Pharm. Bull..

[17] Lee J.Y., Tokumoto M., Fujiwara Y., Hasegawa T., Seko Y., Shimada A., Satoh M. (2016). Accumulation of p53 via down-regulation of UBE2D family genes is a critical pathway for cadmium-induced renal toxicity. Sci. Rep..

[18] Wolff N.A., Abouhamed M., Verroust P.J., Thévenod F. (2006). Megalin-dependent internalization of cadmium-metallothionein and cytotoxicity in cultured renal proximal tubule cells. J. Pharmacol. Exp. Ther..

[19] Fels J., Scharner B., Zarbock R., Zavala Guevara I.P., Lee W.K., Barbier O.C., Thévenod F. (2019). Cadmium complexed with β2-microglubulin, albumin and lipocalin-2 rather than metallothionein cause megalin:cubilin dependent toxicity of the renal proximal tubule. Int. J. Mol. Sci..

[20] Fujishiro H., Hamao S., Isawa M., Himeno S. (2019). Segment-specific and direction-dependent transport of cadmium and manganese in immortalized S1, S2, and S3 cells derived from mouse kidney proximal tubules. J. Toxicol. Sci..

[21] Fujishiro H., Himeno S. (2019). Gene expression profiles of immortalized S1, S2, and S3 cells derived from each segment of mouse kidney proximal tubules. Fundam. Toxicol. Sci..

[22] Fuller S.D., Simons K. (1986). Transferrin receptor polarity and recycling accuracy in “tight” and “leaky” strains of Madin-Darby canine kidney cells. J. Cell Biol..

[23] Smith C.P., Lee W.K., Haley M., Poulsen S.B., Thévenod F., Fenton R.A. (2019). Proximal tubule transferrin uptake is modulated by cellular iron and mediated by apical membrane megalin-cubilin complex and transferrin receptor 1. J. Biol. Chem..

[24] Nielsen R., Christensen E.I., Birn H. (2016). Megalin and cubilin in proximal tubule protein reabsorption: From experimental models to human disease. Kidney Int..

[25] Imura J., Tsuneyama K., Ueda Y., Himeno S., Aoshima K. (2019). Novel Pathological Study of Cadmium Nephropathy of *Itai-Itai* Disease. Cadmium Toxicity—New Aspects in Human Disease, Rice Contamination, and Cytotoxicity.

[26] Satarug S., Vesey D.A., Nishijo M., Ruangyuttikarn W., Gobe G.C. (2019). The inverse association of glomerular function and urinary β_2_-MG excretion and its implications for cadmium health risk assessment. Environ. Res..

[27] Argyropoulos C.P., Chen S.S., Ng Y.H., Roumelioti M.E., Shaffi K., Singh P.P., Tzamaloukas A.H. (2017). Rediscovering beta-2 microglobulin as a biomarker across the spectrum of kidney diseases. Front. Med..

[28] Jin T., Wu X., Tang Y., Nordberg M., Bernard A., Ye T., Kong Q., Lundström N.G., Nordberg G.F. (2004). Environmental epidemiological study and estimation of benchmark dose for renal dysfunction in a cadmium-polluted area in China. Biometals.

[29] Jin T., Kong Q., Ye T., Wu X., Nordberg G.F. (2004). Renal dysfunction of cadmium-exposed workers residing in a cadmium-polluted environment. Biometals.

[30] Liang Y., Lei L., Nilsson J., Li H., Nordberg M., Bernard A., Nordberg G.F., Bergdahl I.A., Jin T. (2012). Renal function after reduction in Cadmium exposure: An 8-year follow-up of residents in Cadmium-polluted areas. Environ. Health Perspect..

[31] Dickson L.E., Wagner M.C., Sandoval R.M., Molitoris B.A. (2014). The proximal tubule and albuminuria: Really!. J. Am. Soc. Nephrol..

