# Supplementary material for: In Vitro Evaluation of the Effects of Cadmium on Endocytic Uptakes of Proteins into Cultured Proximal Tubule Epithelial Cells
Source: Toxics. 2020 Apr 1;8(2):24. doi: 10.3390/toxics8020024 (PMC7356949; doi:10.3390/toxics8020024)
Supplement: Supplementary file 1 [file toxics-08-00024-s001.pdf]

Supplementary Materials: In vitro Evaluation of The Effects of Cadmium on Endocytic Uptakes of Proteins into Cultured Proximal Tubule Epithelial Cells

Hitomi Fujishiro, Hazuki Yamamoto, Nobuki Otera, Nanae Oka, Mei Jinno and Seiichiro Himeno

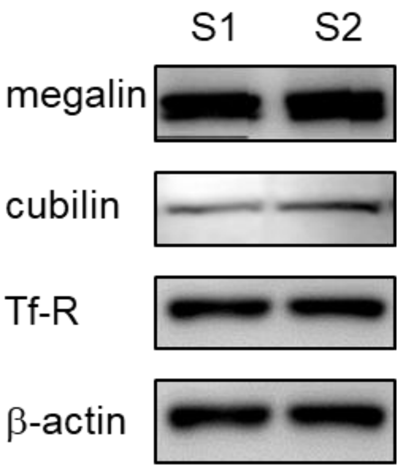

Figure S1. Expression levels of the proteins involved in endocytosis in S1 and S2 cells. Whole cell lysates of S1 and S2 cells were subjected to Western blot analysis. β-actin was used as a loading control. The expression levels of megalin, cubilin, and transferrin receptor (TfR) were almost the same between S1 and S2 cells.

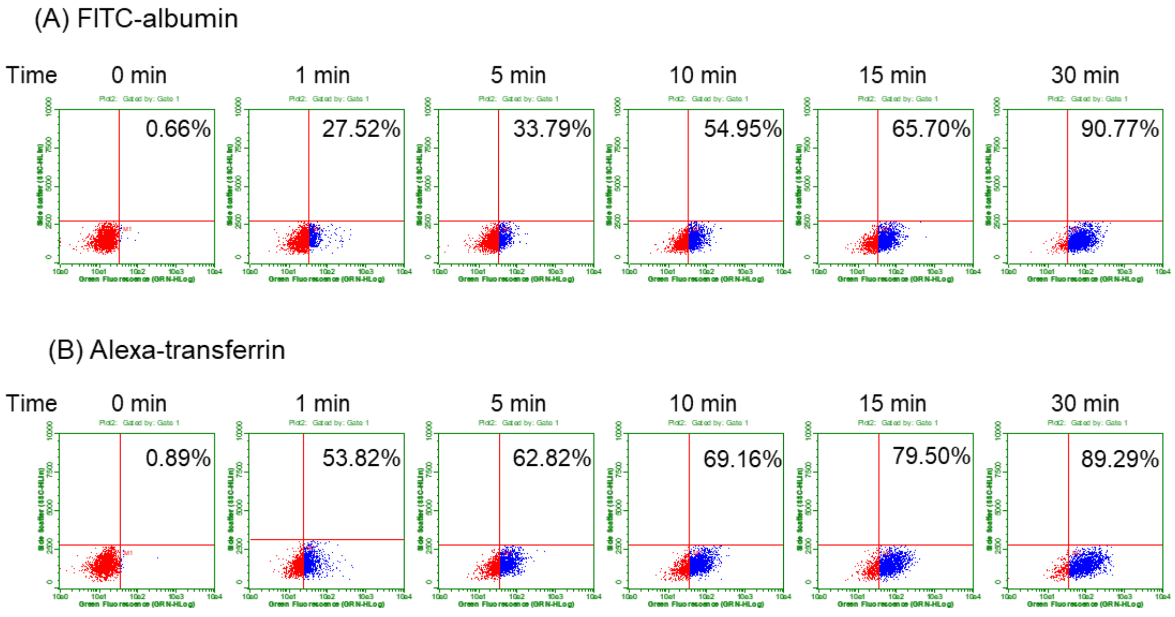

Figure S2. Time-dependent changes in endocytic uptakes of FITC-albumin (**A**) and Alexa-transferrin (**B**). S1 cells were incubated with fluorescent-labeled proteins for 1, 5, 10, 15, and 30 min. The percentages of fluorescent-positive cells were calculated in the same way as in Figure 2. Typical quadrant data were shown here. The cell populations of both proteins in the lower-right, indicative of endocytic uptakes, increased in a time-dependent manner.

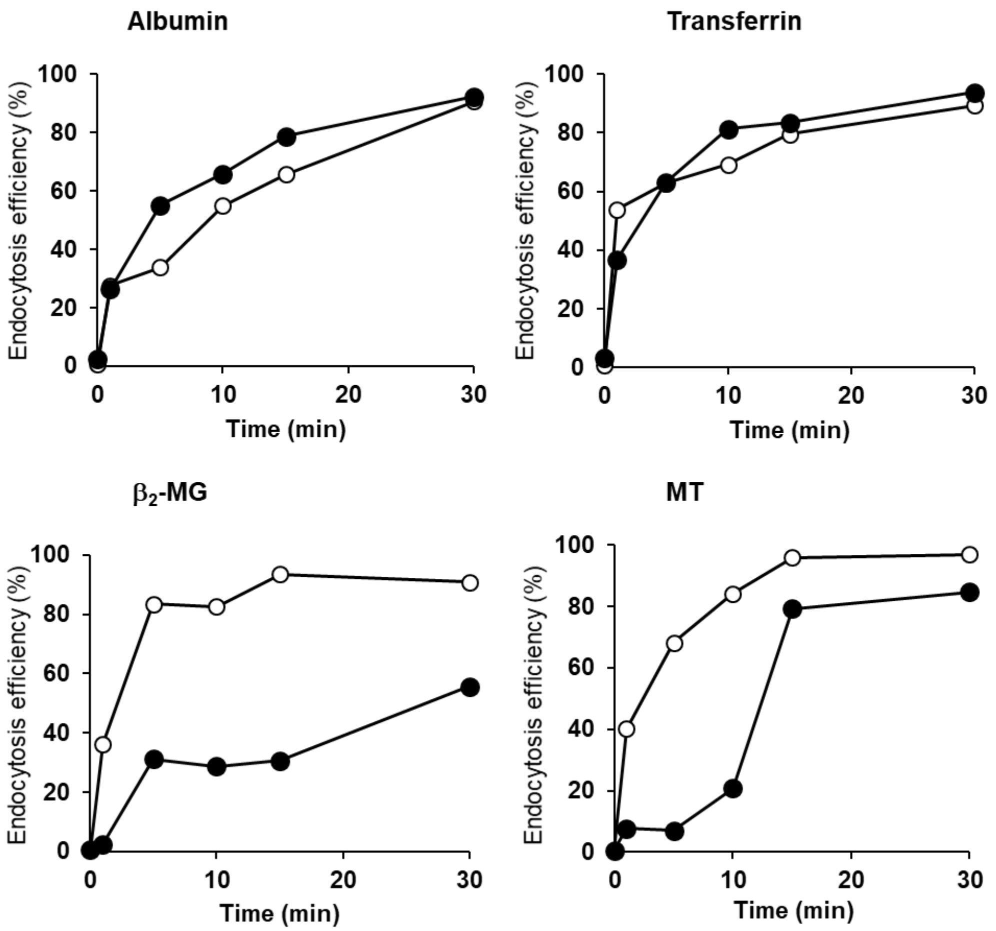

Figure S3. Time-dependent changes in endocytic uptakes of albumin, transferrin, β2-MG, and MT. S1 and S2 cells were incubated with fluorescent-labeled proteins for 1, 5, 10, 15, and 30 min. The fluorescence-positive cells were counted by flow cytometry and expressed as percentages of the total cells. Open and closed circles represent S1 and S2 cells, respectively (*n* = 1–2).

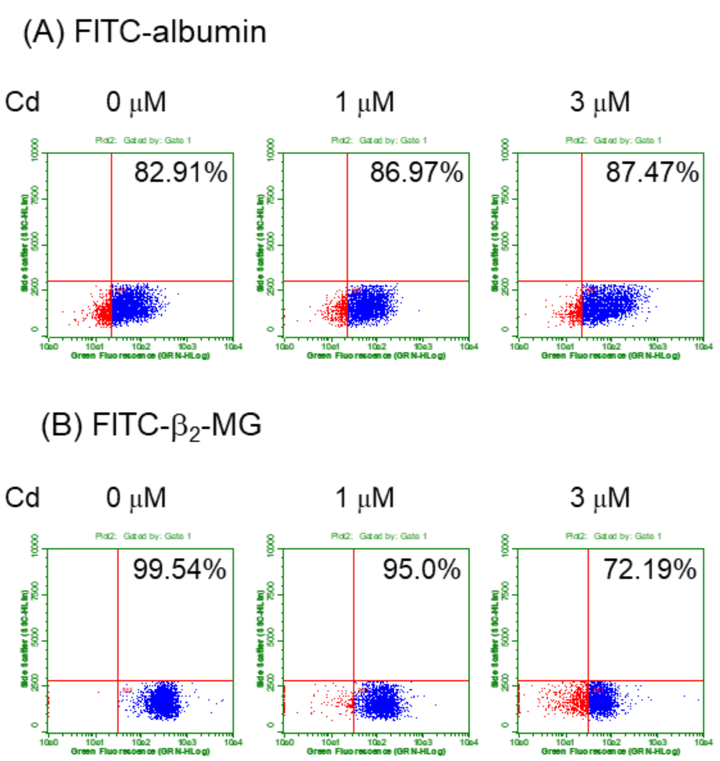

Figure S4. Effects of Cd on the endocytosis efficiencies of albumin and β_2-MG into S1 cells. S1 cells were exposed to $CdCl_2$ for 3 days (1 or 3 μM), washed, harvested, and then incubated with FITC-albumin (**A**) or FITC-β_2-MG (B) for 30 min. The percentages of fluorescent-positive cells were calculated in the same way as in Figure 2. Typical quadrant data were shown here. The cell populations of β_2-MG (**B**), but not those of albumin (A), in the lower-right, indicative of endocytic uptakes, were decreased by exposure to Cd at 3 μM for 3 days.
